# Impact of Green HRM Practices on Environmental Performance: The Mediating Role of Green Innovation

**DOI:** 10.3389/fpsyg.2022.916723

**Published:** 2022-06-14

**Authors:** Yen-Ku Kuo, Tariq Iqbal Khan, Shuja Ul Islam, Fakhrul Zaman Abdullah, Mahir Pradana, Rudsada Kaewsaeng-on

**Affiliations:** ^1^Department of Leisure Industry Management, Commercial College, Chinese Culture University, Taipei, Taiwan; ^2^Department of Management Sciences, The University of Haripur, Haripur, Pakistan; ^3^FAST School of Management, National University of Computer and Emerging Sciences (NUCES), Islamabad, Pakistan; ^4^Department of Management, School of Business, Sulaiman AlRajhi University, AlQassim, Saudi Arabia; ^5^Department of Business Administration, Faculty of Communication and Business, Telkom University, Bandung, Indonesia; ^6^Faculty of Humanities and Social Sciences, Prince of Songkla University, Muang Pattani, Thailand

**Keywords:** green HR practices, innovation, environment, performance, human resource management

## Abstract

Numerous organizations have faced substantial environmental performance challenges resulting from more than a half-century of worldwide industrialization. Grounded in social learning theory and recourse-based view theory, this study explores environmental performance and its impact on employees and industry outcomes. Drawing on a cross-sectional online survey of 500 full-time employees working in the chemical industry in Lahore, Pakistan. The results revealed a significant positive influence of Green HRM practices on employees’ Green innovation as well as on environmental performance. Additionally, significant influences of study variables were recorded on outcomes such as green compensation and reward, green performance management and appraisal, green training and development, and green recruitment and selection. Several key policy insights related to consumer resistance to innovation in low income societies and future research directions are suggested, along with theoretical and practical implications.

## Introduction

Several areas of organizational management have faced substantial environmental performance challenges as an outcome of more than a half-century of worldwide industrialization. In addition, chemicals-related precautionary steps and actions help in the reduction of issues such as climate change, environmental degradation, the release of numerous toxins that pollute the atmosphere and oceans, as well as the release of audio and visual contamination, and maybe even wildlife annihilation (Billig et al., 2022; Darvishmotevali and Altinay, 2022). The worldwide ecosystem and economic and social well-being are threatened by these manufacturing applications and their negative impacts on the environment. This critical scenario needs increased public awareness of environmental or green concerns such as reducing emissions, composting, and renewable technologies such as sunlight, winds, and hydroelectricity. In the recent days, the dangers of environmental challenges have prompted various sectors to concentrate on environmental initiatives, initiate coaching, and retrain their staff in green performance (Johar et al., 2020; Ecer et al., 2021).

Among the most crucial parts of sustainable capabilities is green HRM. Green HRM takes an environmental approach and aims to create a green work environment that encourages workers to perform their jobs in the most environmentally responsible manner. According to current green HRM guidelines and procedures, such as top management involves inspiring employee responsibility toward the environment and team spirit in this region and hiring, satisfying, inspiring self-improvement, and mentoring individuals in accordance with the institution aims (Pimonratanakan and Pooripakdee, 2017; Pham et al., 2019).

Among green HRM practices, green compensation and reward are important practices in which any organization gives rewards and compensation to their employees (Maderazo, 2016; Mandago, 2018). Green compensation and reward have been defined as “a firm should praise and appreciate the efforts of employees in gaining sustainable advantage and give them incentives and rewards, in this way organization will achieve sustainable advantage and employees will also be motivated” (Ahmad, 2015). Another important factor included in Green HRM practices are Green performance and appraisal. It is defined, as “as the extent to which certain employees engage in behavior (actions and activities) and produce results with respect to greening over a certain period” (Bilal and Zia-ur-Rehman, 2017; Ardiza et al., 2021). According to the company, individual production measurement is evaluated on how they are progressing into a greener environment (Mishra, 2017; Ahmad, 2021; Darvishmotevali and Altinay, 2022).

Firms should leverage their internal green social capital to strengthen the benefits that come from their green process innovations. Given that green social capital is a critical factor for speeding up knowledge flow among employees within organizations, firms should take advantage of green social capital by enhancing their ability to communicate and share knowledge among employees to raise awareness of green process innovations. Furthermore, firm managers should employ appropriate human resource management strategies to foster these connections and exchanges, as well as increase trust and social cohesion among employees. Accordingly, if members feel more connected and supported, and they are willing to share information, this can reduce some of the isolation and fragmentation issues that can occur in the pursuit of green process innovation (Xie et al., 2022).

Furthermore, green HRM practices also have important factors such as green training and development. Obaid and Alias (2015) have defined development as the “Development of attitudes, behaviors, knowledge, and skills in the employees that stop the corrosion of environment-related attitudes, skills and knowledge come under the umbrella of training and development.” While, on the contrary, training is defined as “the process of preparing multi-talented individuals for the improvement of instruction necessary for innovations” (Rani and Mishra, 2014; Gill et al., 2021). Similarly, green recruitment and selection have an important part of green HRM practices. Recruitment is defined as “the process of searching prospective employees to apply for the job posting in the organizations and selection is the process of choosing appropriate applicants among the job applicants” (Mwita and Kinemo, 2018). Effective recruitment and selection methods are crucial parts of any organization’s HRM entry point. Thus current research is incremental to integrate green HRM practices with green innovation and environmental performance in organizational context. Such research attempts in past literature special in green HRM literature are scarce. Hence, this study mainly contributes toward green HRM literature.

This study has advanced by bridging the research gap between key variables and linking the above HRM practices with green innovation and environmental performance. In past research, the environmental performance has been defined as “combining with the organization’s external and internal management to achieve overall economic performance like increasing market share and brand image and improving the performance of their strategic partners by making eco-friendly environment and reducing risks associated with the environment” (Van Hock and Erasmus, 2000). In addition to that green innovation using as a mediating variable, which was defined as “the performance of hardware and software involved in the innovation that a company carries out in relations to green products or processes, including the innovation in technologies that are involved in energy saving, pollution prevention, waste recycling, green product designs, or corporate environmental management” (Chen et al., 2006).

Finally, the present study is incremental due to conceptual grounding in social learning theory and recourse-based view theory. Social learning theory (SLT) has been defined as “how both environmental and cognitive factors interact to influence human learning and behavior” (Li et al., 2019). Recourse-based view theory explains resources as “valuable when they enable a firm to conceive and implement strategies that improve its efficiency and effectiveness” (Pohjola, 2002). The integration of both theories explains how HRM practices can help utilize existing unique resources by learning environmental practices to bring sustainable competitive advantage to the organization.

The study utilizes a developing country context of the Pakistani chemical industry located in the industrial hub city of Lahore. A recent survey about the environmental quality index about Lahore city has raised a question mark about the alarming situation in the second most populous city in the country (Islam et al., 2021a). Thus, the study becomes most relevant to the contextual case of how green HRM practices can lead an organization towards green innovation, and through green innovation, these HRM practices are linked with environmental performance. The contextual case of a populous city resembles with settings of most of the populous cities across the globe in general and the developing world in specific. Thus current study’s choice of setting will bring several generalizable key policy insights for administrators and urban planners to enhance environmental performance. Finally, the current study aims to shed light to explore the green compensation and reward, green performance and appraisal, green training and development, and green recruitment and selection impact on environmental performance. Additionally, green innovation impact and mediation of green innovation has been explored between green HR practices and environmental performance.

## Literature Review

This research focused on green HRM practices, such as green compensation and reward, green performance and appraisal, green training and development, and green recruitment and selection, and its impact on environmental performance through a mediated link of green innovation. The present study has established its own social learning theory and recourse-based view theory.

The resource-based view firm hypothesis was developed in the early 1980s and became more widely known in the 1990s. This theory plays an important role in green HRM practices that are “valuable,” “rare,” “imperfectly imitable,” and “non-substitutable.” In addition, social learning theory can have cognitive interaction with human behavior and learn through environmental awareness campaigns by the human resource department and top management of the organization. Both theories also involved the rare, valuable, and unique resources coupled with rewards and compensation through learning, awareness, and motivation. Combining two diverse theories helps to understand how awareness campaigns coupled with rewards and motivational factors focused on human resources may bring sustainable competitive advantage for the firms engaged in green HRM practices.

### Green Compensation and Reward and Environmental Performance

Green compensation and rewards is a monetary and nonmonetary incentive program intended to capture, preserve, and encourage people to support green environmental priorities (Mandago, 2018). The following are the dimensions of green compensation and rewards: (1) rewards for skill; (2) cognitive and interpersonal; (3) sustainable technology appreciative inquiry; and (4) rewards for green, and sustainable behavior acceptability (Ahmad, 2015). The previous literature also reported that employee loyalty to environmental sustainability programs was boosted when they were granted cash to bring on activities related to environmental responsibilities. Green rewards and compensation were proven successful by Berrone and Gomez-Mejia (2009), who researched 469 US businesses that operate in high levels of contaminants industries, which was a similar setting to the current study. Employee satisfaction with green rewards and support in establishing environmental performance is stressed in green ability to do the job (Jabbar and Abid, 2015; Silahtaroglu and Vardarler, 2016; Silva and Madushani, 2017).

On the contrary, green rewards contribute to the best quality of work-life, which considerably improves environmental performance (Jabbar and Abid, 2015). Similar findings have been reported in work performance with the environment is influenced by green rewards and compensation (Renwick et al., 2013). The use of incentives and acknowledgment based on environmental performance positively impacts staff willing to try out green projects (Rawashdeh, 2018). These conditions suggest that green and environmentally focused compensation and rewards are expected to be directly linked with the organization’s environmental performance. Hence, the following hypothesis is suggested:

*H1*: Green compensation and reward are positively associated with environmental performance.

### Green Performance Appraisal and Environmental Performance

The use of organization-wide measures for measuring resource consumption and waste is essential for long-term environmental performance. A conceptual model for sustainable development, which tracks and audits the production and use of assets, is also required for high achievement (Ojo et al., 2020; Prakash and Das, 2022). Another important factor included in green HRM practices is green performance and appraisal. According to the company, individual production measurement is evaluated on how they are progressing into a greener environment (Mishra, 2017; Darvishmotevali and Altinay, 2022).

Measurement techniques in performance management are an important green human resource management strategy since they allow people to obtain immediate feedback on their environmental practices (Chen et al., 2015). As a result, staff can evaluate their effectiveness in relation to the expected environmental performance. According to Govindarajulu and Daily (2004), giving workers timely information on their environmental performances might grab their attention and encourage them to engage in the desired outcome. According to the research, people are more inclined to alter their habits in response to the critical success factors evaluated through green performance management (Darvishmotevali and Altinay, 2022).

Although extending the previous literature and establishing the social learning theory and recourse-based view theory, a direct association is expected between green performance appraisal and environmental performance; as a result, the current research suggests the following hypothesis:

*H2*: Green performance appraisal is positively associated with environmental performance.

### Green Training and Development and Environmental Performance

Employees’ training is critical in mobilizing them with the skills and expertise needed to make informed decisions about green HRM practices (Ojo et al., 2020). As a result, they will be motivated to implement green; furthermore, green HRM practices also have another important factor: Green training and development. Training is defined as “the process of preparing multi-talented individuals to improve instruction necessary for innovations” (Rani and Mishra, 2014; Gill et al., 2021). In green HRM practices, training includes providing staff with core competencies such as teaching them how to gather trash information and increasing the company’s standard of efficiency and environmental competency (Jabbar and Abid, 2015; Ojo et al., 2020). Workers’ desire to contribute to environmentally friendly efforts requires environmental training (Mishra, 2017; Mandago, 2018). Training may help individuals educate about occupational difficulties and transitions, enhance and improve their abilities, and drive them to fulfill the task (Rani and Mishra, 2014). Extending the previous literature, current research expects a direct link between green training and development and environmental performance. Hence, the following hypothesis is suggested:

*H3*: Green training and development is positively associated with environmental performance.

### Green Recruitment and Selection and Environmental Performance

Employment registrations can be handled *via* the website as part of the green recruitment and selection process, which includes employing environmentally conscious individuals without papers. Interviews can be done over the phone or *via* the internet (Renwick et al., 2008). Green recruiting initiation supports job performance in long-term performance assessment and educating employees about green corporation efforts such as lowering waste and environmental damage. This, in turn, helps to improve environmental performance (Nayak and Mohanty, 2017).

Green recruitment and selection have an essential part of green HRM practices. Effective recruitment and selection methods are crucial parts of any organization’s HRM entry point. The real importance is enhanced by hiring and selection procedures. By recruiting and keeping resembling employees, green recruitment highlights an organization’s willingness to cooperate to benefit the environmental performance (Masri and Jaaron, 2017). Green recruitment, apart from its influence on the attraction of internal recruitment to companies, displays the organization’s environmental performance. Hence, the current study suggested the following hypothesis:

*H4*: Green recruitment and selection is positively associated with environmental performance.

### Green Innovation and Environmental Performance

According to past research, the effectiveness of sustainable products, green management, and quality advancement and the inclusion of ecological and financial green practices into company efficiency and overall improvement all have an impact on environmental performance (Darnall et al., 2008; Chen et al., 2015; Dubey et al., 2015; Ardiza et al., 2021). Another important past research reported that green innovation is connected to a corporate methodology is based, and that green innovation boosts environmental performance (Chen et al., 2006; Kammerer, 2009). Moreover, green production process improvement reduces a company’s unfavorable environmental effect and improves its economic and personal performance by reducing material wastage (Weng et al., 2015). According to past research, green innovation should not be viewed as a company’s reaction to customer concerns but rather as going through a process aim and practice to improve environmental performance. It was estimated that green processes and new green product developments are essential organizational assets that the business will deploy to enhance its environmental performance and obtain trust with relevant parties *via* the RBV (Dubey et al., 2015). The mentioned new green product developments are associated with emerging green innovation in the organizational context and are expected to be directly linked with the organization’s environmental performance. Thus, the following hypothesis is suggested:

*H5*: Green innovation is positively associated with environmental performance.

### Mediating Role of Green Innovation

Environmental damage has been a constant source of influence on industry to limit the environmental impact of its operational and production activities. Competitiveness in today’s turbulent marketplace significantly affects any company’s success and sustainability, and expectations for outstanding profitability and customers’ perceptions play an important role in the overall image. As a result, organizational resources, production strategies, and efficiency are critical in dealing with environmental issues, forcing the organizations to prioritize green innovation. Furthermore, business assets alone are insufficient for green innovation, new tactics, techniques, and competencies (Pacheco et al., 2018). Green innovation, without a doubt, has a significant effect on long-term environmental performance (Zhu et al., 2017).

In previous studies, researchers used green innovation as a mediating variable with financial resources and financial performance (Mwita and Kinemo, 2018), talent capability and competitive advantage business analytics, and capability management (Waqas et al., 2021). Some of the past research also investigated green innovation with green opportunities, environmental performance (Berrone and Gomez-Mejia, 2009), green ability, green motivation (Darnall et al., 2008), environmental strategy, and CSR (Kraus et al., 2020; Ahmad et al., 2021). Thus, leading the way forward to theoretically propose based on RBV theory and social learning theory and further investigate this mediated phenomenon empirically between HRM practices and environmental performance. Hence, the following hypothesis is suggested:

*H6*: Green innovation mediates the relationship between green HRM practices (i.e., compensation and reward, green performance and appraisal, green training and development, and green recruitment and selection) and environmental performance.

## Research Methodology

### Participants and Procedure

**Figure 1:**
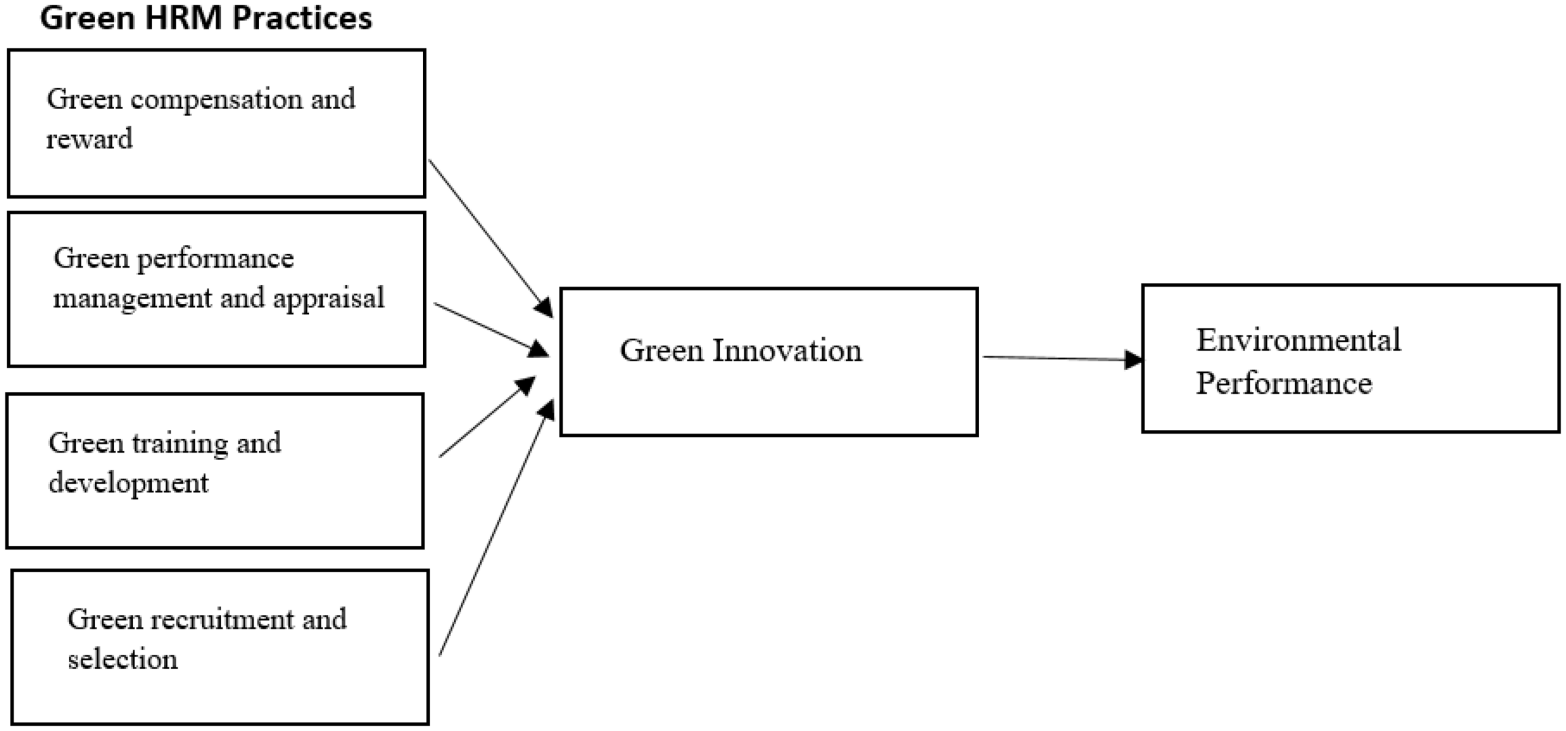
Theoretical framework.

Based on a thorough literature analysis and theoretical groundings such as social learning theory and recourse-based perspective theory, a coherent conceptual framework has been developed, as shown in Figure 1, and hypotheses were proposed in this research. The survey’s target population was permanent employees of the Ittehad Chemicals industry in Lahore; this study was chosen because of its success and reputation in Pakistan related to green environment initiatives and green human resource practices. Its nature of dealing with hazardous material and environmental consciousness in this industry related to green initiatives are main factors for choosing Ittehad Chemicals as study setting. A letter outlining the research goals was written to their management to get their official consent and approval to participate in the study. Their employees’ email addresses were gathered from targeted chemical industry management officials who agreed to participate in the survey after receiving an initial approval letter from the researchers’ institution. The confidentiality of organizations’ and employees’ identities were protected and ensured that at no point throughout the study project and even after the project any personal information would not be shared with a third party for any purpose.

To determine the appropriate sample size for the study a similar recent study was taken as a reference in similar context to justify the appropriateness of sample (Islam et al., 2022). A total of 700 participants were contacted, with 540 agreeing to participate in a voluntary online survey. Authors sent online questionnaires *via* email and social media links; due to strict COVID-19 protocols, no physical contact was allowed. The official language of the survey was English, as it was ensured that only employees who are educated and have good expertise in the English language should take part in this survey. The survey began on November 10, 2021, and the authors received 540 completed questionnaires by January 20, 2022. Multiple follow-ups and reminders were also given during this time for better response. Due to unengaged and partially filled responses, 40 responses were eliminated after the initial evaluation based on partially filled or unengaged response observed by study authors. So the total response count was 500, with 71% of active respondents.

The strategy to perform analysis was initially demographic factors and then PLS software was used to test the hypothesized associations.

### Measures of the Study

1. A 23-item questionnaire was created to investigate the impact of environmental performance on green innovation and green HRM practices.
2. A 3-item scale was adopted to determine the green compensation and reward (Haldorai et al., 2022). Sample items include “Employees are rewarded for making suggestions for improvement on environmental programs” and “Employees who have achieved or surpassed their environmental goals are rewarded with bonus pay or other monetary awards.”
3. A 3-item scale was adopted to determine the green performance and appraisal (Haldorai et al., 2022). Sample items include “Environmental goals and objectives are implemented in this organization for all employees” and “Contributions to environmental management are assessed.”
4. A 5-item scale was adopted to determine green training and development (Haldorai et al., 2022). Sample items include “This organization offers ecological training for all employees” and “In this organization, environmental training is a priority.”
5. A 4-item scale was adopted to determine green recruitment and selection (Haldorai et al., 2022). Sample items include “This organization is very particular about mainly recruiting and selecting employees with environmental concerns, knowledge, and attitude” and “In the recruitment process, our organization focuses on applicants with environmental insights, attitude, and concerns.”
6. A 3-item scale was adopted to determine green innovation (Tseng et al., 2013). Sample items include “Investment in green equipment and technology” and “Implementation of the comprehensive material saving plan.”
7. A 5-item scale was adopted to determine the environmental performance (Sobaih et al., 2020). Sample items include “Environmental activities significantly improved my organization’s reputation” and “Environmental activities significantly reduced waste within the entire value chain process.” In all the above scales, the responses were tapped on “a 5-point Likert scale ranging from 1 = Strongly Disagree to 5 = Strongly Agree.”

## Data Analysis and Results

### Measurement Model

SmartPLS3 was applied to assess the measurement and structural model. The simulation analysis in the study revealed that respondents’ gender, designation, and marital status positively impacted their perceptions about green innovation and environmental performance; therefore, all these three demographic characteristics were controlled during the study. The demographic detail of this study respondents is presented in Table 1.

**Table 1:** Demographic profile.

Demography	Description	No. of responses	%
Gender	Male	375	75.0
	Female	125	25.0
Marital status	Married	320	64.0
	Not married	180	36.0
Designation	Manager	50	10.0
	Co-supervisor	111	22.2
	Employees	339	67.8

Furthermore, using the measurement model, “Cronbach’s (CA)” and “composite reliability (CR)” were computed to evaluate the measurements’ consistency (Henseler et al., 2015). CA and CR for all research constructs were greater than 0.7, indicating that they meet the required reliability criterion (Sarstedt et al., 2017). Then, to determine the constructs’ convergent validity, factor loadings and average variance extracted (AVE) were determined (Sarstedt et al., 2017). All factor loading of the study constructs was over the minimal criterion of 0.70 in both investigations, and AVE was above 0.50 (Henseler et al., 2015). A full description of the article’s validity and reliability measurements are given in Table 2.

**Table 2:** Composite reliability, Cronbach’s alpha, and AVE values.

Constructs/items	Cronbach’s alpha	AVE	CR	AVE SQRT
Environmental performance	0.868	0.909	0.903	0.652
Green innovation	0.730	0.790	0.828	0.618
Green compensation and reward	0.707	0.726	0.836	0.630
Green performance and appraisal	0.701	0.705	0.834	0.626
Green recruitment and selection	0.791	0.695	0.864	0.614
Green training and development	0.826	0.831	0.874	0.591

CR, composite reliability and AVE, average variance extracted.

**Figure 2:**
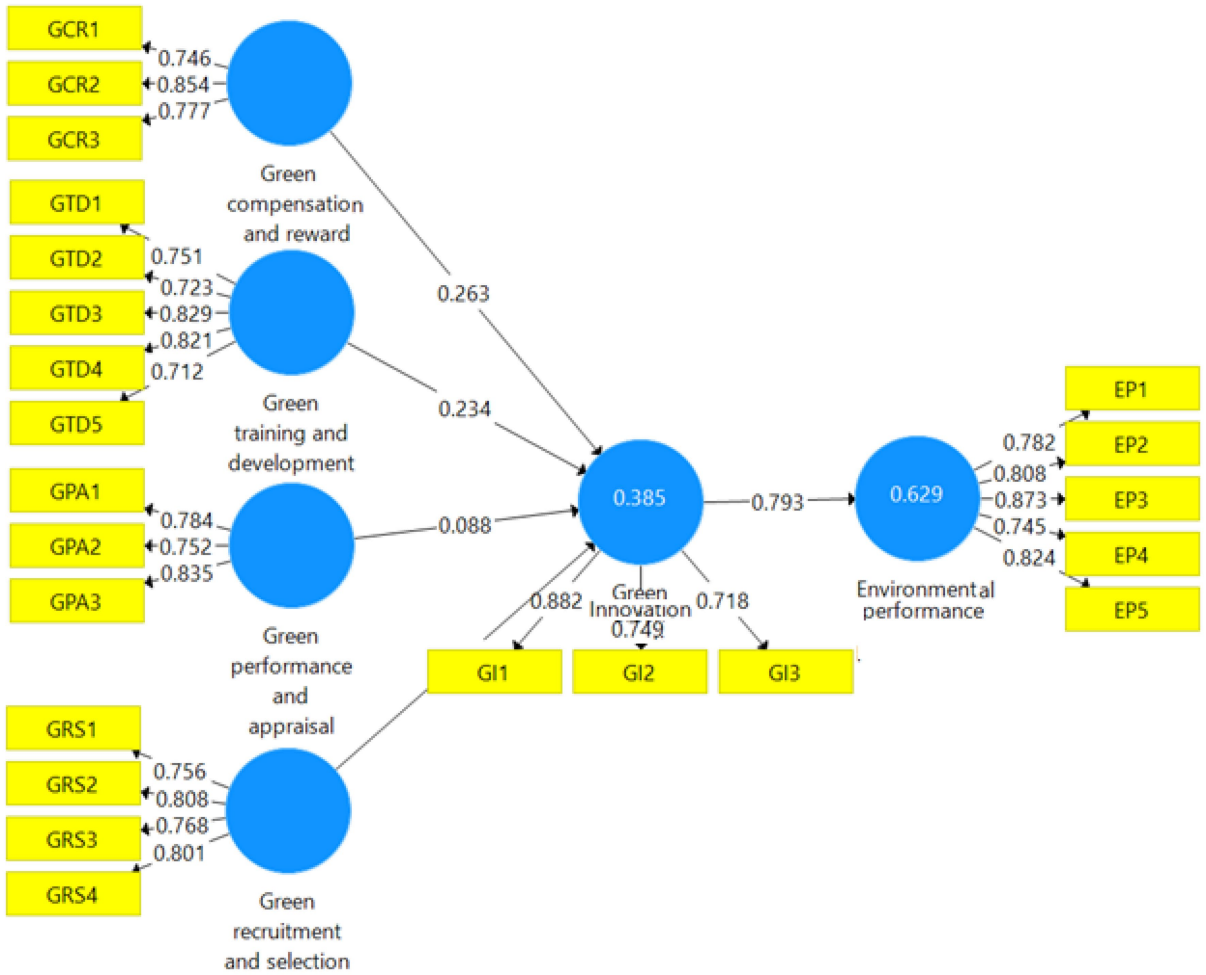
PLS algorithm.

Moreover, to establish the discriminant validity of all the study constructs, Fornell and Larcker (1981) defined discriminant validity as “the extent to which a particular latent variable differs from other latent variables.” By analyzing the correlation between the latent construct and the square root of AVE, discriminant validity was determined (Sarstedt et al., 2017). According to Sarstedt et al. (2017), the use of latent variables retrieved with a value of 0.50 or more is recommended for determining discriminant validity. According to Sarstedt et al. (2017), discriminant validity is indicated when the square root of AVE is greater than the value of latent variables. The PLS algorithm is presented in Figure 2. Similarly, the values of discriminant validity are presented in Table 3.

**Table 3:** Discriminant validity.

	EP	GI	GCR	GPA	GRS	GTD
EP	0.808					
GI	0.793	0.786				
GCR	0.505	0.476	0.794			
GPA	0.231	0.270	0.193	0.791		
GRS	0.535	0.468	0.367	0.183	0.786	
GTD	0.588	0.462	0.397	0.348	0.340	0.769

EP, environmental performance; GI, green innovation; GCR, green compensation and reward; GPA, green performance and appraisal; GRS, green recruitment and selection; and GTD, green training and development.

### Assessment of Structural Model

#### Hypothesis Testing

Regarding the consideration of measurement model clear links, this part focuses on the structural model, as described by Sarstedt et al. (2017). A structural model is used in the hypothesized model to highlight the relationship’s reliance on one another. The structural model in PLS gives an inner modeling study of the direct relationship between the proposed hypotheses and their *t*-values and regression coefficient. In regression analysis, an indirect effect is the same as a standardized beta value; according to (Henseler et al., 2015), *t*-values and beta values of the regression coefficient are used to determine significance. *T*-values larger than 1.64 are deemed statistically significant, according to Hair et al. (2017), which are then utilized to make choices on the purposed hypothesis. There are two main purposes of studying the model: examining direct relationships and testing predicted relationships between components using a structural model. Six hypotheses are examined in this study. According to Ramayah et al. (2018), SmartPLS 3.0 output findings include path coefficients, *t*-values, *p*-values, and standard errors. The researcher used them to determine whether the hypothesis was supported or not, and the results are presented in Table 4.

**Table 4:** Hypothesis testing.

	*B*-value	Sample mean	Standard deviation	*T*-value	*p*	
GI→EP	0.793	0.796	0.014	6.664	0.000	Accept
GCR→GI	0.263	0.261	0.057	4.616	0.000	Accept
GPA→GI	0.288	0.290	0.054	2.162	0.001	Accept
GRS→GI	0.274	0.275	0.056	4.908	0.000	Accept
GTD→GI	0.234	0.235	0.065	3.586	0.000	Accept

EP, environmental performance; GI, green innovation; GCR, green compensation and reward; GPA, green performance and appraisal; GRS, green recruitment and selection; and GTD, green training and development.

#### Assessment of R2

**Figure 3:**
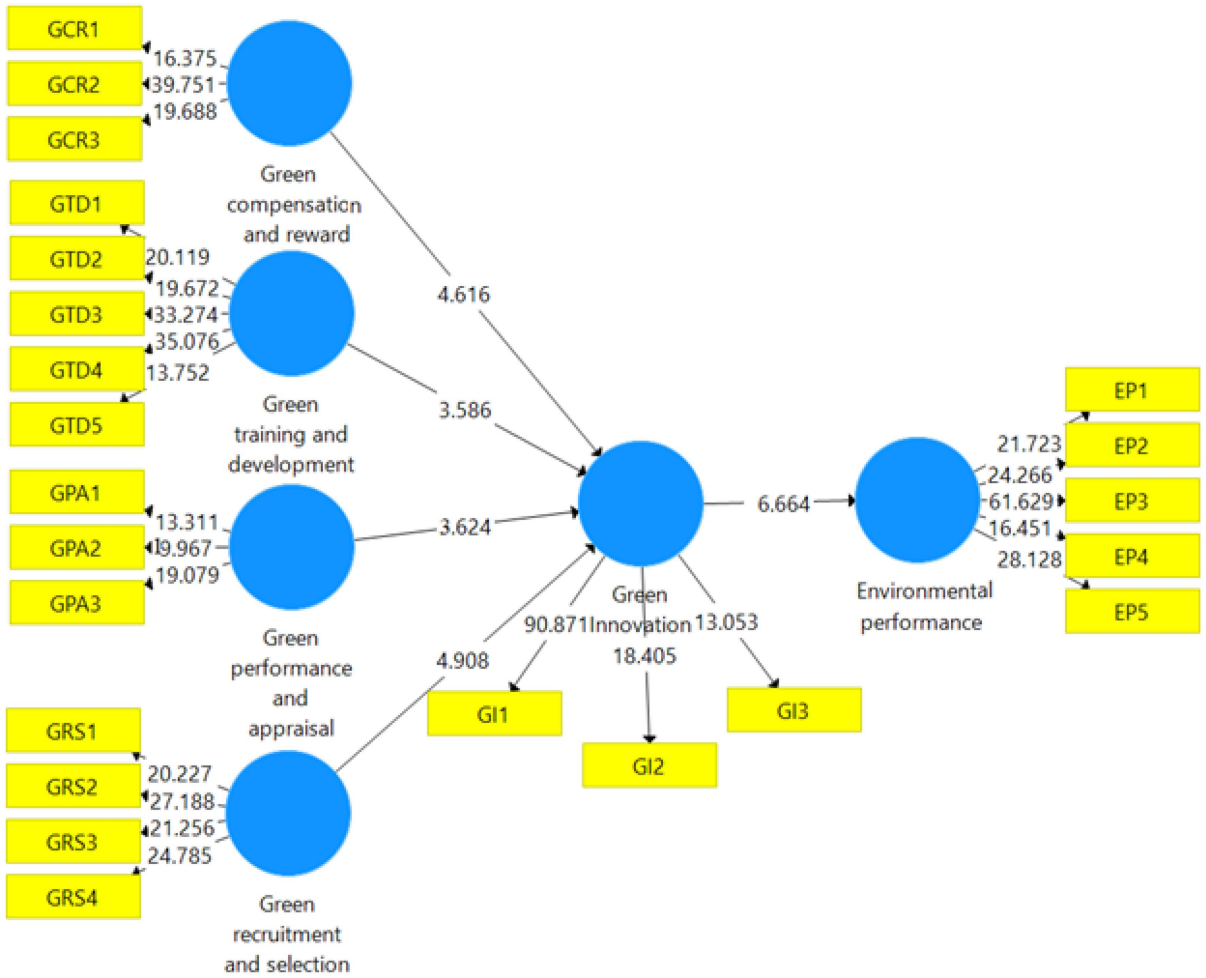
PLS bootstrapping.

The second stage in analyzing a structural model is determining the coefficient of determination (Hair et al., 2011). The variance in endogenous constructs caused by external constructs is represented by the coefficient of determination (Hair et al., 2011). Rigdon (2012) stated that the coefficient of determination is also recognized as a sample’s predictive power. If the coefficient of determination is greater, the predictive power of the sample is also greater. The value of R^2^ ranges from zero to one. Moreover, Chin (1998) recommended that the R^2^ of 0.13 is considered weak, 0.33 is considered moderate, and 0.67 is considered strong. The coefficient of determination for endogenous constructs is given in Table 5. The PLS bootstrapping is presented in Figure 3.

**Table 5:** Assessment of *R* square.

	*R* ^2^
Environmental performance	0.629
Green innovation	0.385

## Discussion, Implications, Limitations, and Future Research Directions

### Findings of the Study

The results of the current study reveal that positive associations of green compensation and reward, green recruitment and selection, green performance and appraisal, and green training and development influence environmental performance through green innovation impact on environmental performance. Results showed the significant effect of green performance and appraisal on environmental performance. These results are in line with the findings of Govindarajulu and Daily (2004) who provided timely information about socially desirable behaviors that helps improve employee environmental performance. Green training and development have a significant impact on environmental performance. In green HRM practices, training includes providing staff with core competencies such as teaching them how to gather trash information and increasing the company’s standard of efficiency and environmental competency (Jabbar and Abid, 2015; Ojo et al., 2020). Another result showed that green compensation and reward significantly impact environmental performance. Employee satisfaction with green rewards and compensation in establishing environmental performance is stressed in green ability to do the job (Jabbar and Abid, 2015). Green recruitment and selection significantly impacted environmental performance in this study results. By recruiting and keeping resembling employees, green recruitment highlights an organization’s willingness to cooperate for the benefit of the environmental performance (Masri and Jaaron, 2017). The results further show positive associations between green compensation and reward, green recruitment and selection, green performance and appraisal, and green training and development, which influence environmental performance, with green innovation influencing environmental performance.

Furthermore, the findings of mediation results revealed strong support for the purposed hypotheses that green innovation mediated between green HRM practices and environmental performance. These findings were consistent with similar mediated research explorations in recent literature and also in similar context (Islam et al., 2021a, 2022).

### Theoretical Implications

There are several theoretical implications of the current study. First, as established in the theories such as social learning theory and recourse-based view theory, the current study suggests that positive associations of green compensation and reward, green recruitment and selection, green performance and appraisal, and green training and development influence environmental performance through the mediated link of green innovation. Second, much of the academic research on the RBV has been conducted in industrialized countries, but little is recognized about the RBV beyond this environment (Vargas-Halabí et al., 2017). This research is incremental due to its choice of study context and RBV approach to environmental performance and green innovation. Another major theoretical contribution of this study is combining HRM practices with green innovation and environmental performance. Such studies are rare in the literature related to innovation and HRM practices. Thus current research opened several new avenues of theoretical integration for future scholars in innovation management, HRM, and environmental science. Integration of theories from all three domains together to come up with solid theoretical foundations is the major advance of this research that will help bridge the theory gap among these three domains of research. The use of social learning theory and its integration with RBV as well as green HRM concepts along with green innovation and environmental performance is a major contribution and theoretical advance pitched by current research.

### Practical Implications

The current study also brings valuable insights for policymakers and practitioners in multiple ways. First, the current study shows that green innovation is the most important factor used in this study to elaborate the concept of environmental performance with green HRM practices. It brings key insights to HR managers and top management of environmentally conscious sectors and consumer markets. These days, organizations continuously seek ways to influence environmentally conscious consumers through their environmentally friendly campaigns. In such a competitive environment, practitioners may learn from the approach discussed in this research. Second, environmental performance becomes a priority for top management and authorities, so developing industries’ environmental performance methodological approach to minimize waste, contamination, pollutants, preserve freshwater, electricity, and non-sustainable minerals contributes to improved environmental performance. Finally, they improve environmental performance by reducing chemicals wastage, avoiding polluted water draining into oceans and rivers, and controlling polluted air of industry. Industry human resource managers may set goals and exemplary measures to accomplish green goals to empower their workers. Green campaigns with motivation could be a wonderful way to encourage people to go green. Green HRM might be a more visible aspect of a company’s responsibilities and CSR measures. The current study’s findings showed that green innovation and environmental performance are the most important factors for the success of any organization working in a green consumer environment. In developing countries like Pakistan, customer resistance in the form of usage barriers can be lowered by use of one of three strategies developing a systems perspective, integrating the innovation with preceding activity, and mandating usage through governmental legislation. Value barriers can be lowered through one of three strategies: improving product performance, positioning the product successfully, and reducing price to the consumer through cost efficiency (Islam et al., 2021a). Risk barriers can be reduced with the following strategies: using a well-known brand name, eliciting endorsements and testimonials from users, and facilitating product trial. Traditional barriers can be overcome by educating the consumers and/or using agents; in some instances, the marketing firm may just have to respect the traditions and norms of the users and realize that coping with the situation is the best possible solution (Islam et al., 2021b). Three strategies are available for countering the image barrier: borrow a good image (such as a known brand name), make fun of the negative image that currently exists, or create a unique image for the innovation. Each of these strategies may be classified into one of five types: product strategy, communication strategy, pricing strategy, market strategy, and coping strategy.

### Limitations and Future Research Directions

As environmental consciousness has grown, the notion of “going green” has been a major focus of several organizations. For obtaining a high degree of environmental performance, green factors such as support from management and green intangible resources are critical. In addition to various strengths, the current study also has some limitations that must be addressed in the future. First, the current research has been conducted among the users of a chemical industry located in Lahore, Pakistan, making the findings specific to the chemical industry. Future studies may consider a comprehensive sample from other industries and may also include users/consumers of other industries, like food, fabric, hotels, etc., to come up with findings that can be generalized. Second, although this study was conducted to examine the cross-sectional method, the data were collected at one point in time. Future scholars may adopt longitudinal research design for better causality. In the current study, researchers could not tap the moderating effect of several key constructs due to time and resource constraints. Researchers are encouraged to focus on moderating effects for more significant results toward theory and practice in future studies. Third, this study outcome was only environmental performance mediated by green innovation. In the future, researchers may also add sustainable economic and social performance and environmental performance. Finally, current research only uses a selected population from a developing country context. In the future, researchers may collect data from many employees in a developed country context. A comparative approach toward green HRM practices in developing and developed setting maybe another significant area of future research. Green HRM was not considered as dimensional in this study is a limitation due to theoretical conceptualization of this research focused more on green innovation and environmental performance. Thus future studies may consider it as dimensional construct and test mediation accordingly.

## Conclusion

Grounded in the RBV approach, this study integrated constructs from three different research fields: environmental science, innovation management, and human resource management. This integration based on theoretical grounding is a major advance to the body of knowledge. Besides this unique conceptualization, the current study adopted a study context of a developing country and the organizational context of the chemical industry, which is more sensitive to green innovation and consumer resistance to hazardous environmental products. Thus making this research a unique contribution from a contextual point of view. The findings also confirmed how green HRM practices significantly influenced green innovation and, in turn, predicted the organization’s environmental performance. These findings bring key policy insights for researchers in consumer resistance to innovation, consumer adoption to innovation that how green innovation may help the companies gain sustainable competitive advantage by investing in their human resources. This research helped bridge the gap between various research domains and opened several new avenues for future research.

## Data Availability Statement

The raw data supporting the conclusions of this article will be made available by the authors, without undue reservation.

## Author Contributions

Y-KK and TK developed the idea and helped to refine theoretical framework as well theory grounding and overall proof editing and improving the final paper. SI helped in data collection. FA helped to perform analysis and write-up results section. MP helped in writing literature review and hypotheses development, while RK-o has written discussion, conclusion, and implication sections. All authors contributed to the article and approved the submitted version.

## Conflict of Interest

The authors declare that the research was conducted in the absence of any commercial or financial relationships that could be construed as a potential conflict of interest.

## Publisher’s Note

All claims expressed in this article are solely those of the authors and do not necessarily represent those of their affiliated organizations, or those of the publisher, the editors and the reviewers. Any product that may be evaluated in this article, or claim that may be made by its manufacturer, is not guaranteed or endorsed by the publisher.
